# Establishing a clinical phenotype for cachexia in end stage kidney disease – study protocol

**DOI:** 10.1186/s12882-018-0819-3

**Published:** 2018-02-13

**Authors:** Joanne Reid, Helen R. Noble, Gary Adamson, Andrew Davenport, Ken Farrington, Denis Fouque, Kamyar Kalantar-Zadeh, John Mallett, C. McKeaveney, S. Porter, David S. Seres, Joanne Shields, Adrian Slee, Miles D. Witham, Alexander P. Maxwell

**Affiliations:** 1 School of Nursing and Midwifery, Queen’s University Belfast Medical Biology Centre, 97 Lisburn Rd, Belfast, BT9 7BL UK; 2 School of Psychology, Ulster University Magee Campus, Londonderry, BT48 7JL UK; 3 UCL Centre for Nephrology, Royal Free Hospital, University College London, Pond Street, London, NW3 2QG UK; 4 East and North Hertfordshire University NHS Trust, Lister Hospital, Coreys Mill Lane, Stevenage, SG1 4AB UK; 5 Department of Nephrology, Université de Lyon, UCBL, Carmen, Centre Hospitalier Lyon Sud, F-69495 Pierre-Bénite, France; 6 Division of Nephrology and Hypertension, Pediatrics and Public Health, University of California, Los Angeles, USA; 7 Department of Social Sciences and Social Work, Bournemouth University, Poole, UK; 8 Columbia University Medical Centre, NY Presbyterian Hospital, New York, USA; 9 Regional Nephrology Unit, Belfast City Hospital, Belfast HSC Trust, Belfast, UK; 10 UCL, Faculty of Medical Sciences, Gower St, Bloomsbury, London, WC1E 6BT UK; 11 School of Medicine, Ninewells Hospital, Dundee, DD1 9SY UK; 12 School of Medicine, Dentistry and Biomedical Sciences, Queens University Belfast and Regional Nephrology Unit, Belfast City Hospital, Belfast HSC Trust, Belfast, UK

**Keywords:** End-stage kidney disease, Cachexia, Phenotype, Definition, Longitudinal

## Abstract

**Background:**

Surveys using traditional measures of nutritional status indicate that muscle wasting is common among persons with end-stage kidney disease (ESKD). Up to 75% of adults undergoing maintenance dialysis show some evidence of muscle wasting. ESKD is associated with an increase in inflammatory cytokines and can result in cachexia, with the loss of muscle and fat stores. At present, only limited data are available on the classification of wasting experienced by persons with ESKD. Individuals with ESKD often exhibit symptoms of anorexia, loss of lean muscle mass and altered energy expenditure. These symptoms are consistent with the syndrome of cachexia observed in other chronic diseases, such as cancer, heart failure, and acquired immune deficiency syndrome. While definitions of cachexia have been developed for some diseases, such as cardiac failure and cancer, no specific cachexia definition has been established for chronic kidney disease. The importance of developing a definition of cachexia in a population with ESKD is underscored by the negative impact that symptoms of cachexia have on quality of life and the association of cachexia with a substantially increased risk of premature mortality. The aim of this study is to determine the clinical phenotype of cachexia specific to individuals with ESKD.

**Methods:**

A longitudinal study which will recruit adult patients with ESKD receiving haemodialysis attending a Regional Nephrology Unit within the United Kingdom. Patients will be followed 2 monthly over 12 months and measurements of weight; lean muscle mass (bioelectrical impedance, mid upper arm muscle circumference and tricep skin fold thickness); muscle strength (hand held dynamometer), fatigue, anorexia and quality of life collected. We will determine if they experience (and to what degree) the known characteristics associated with cachexia.

**Discussion:**

Cachexia is a debilitating condition associated with an extremely poor outcome. Definitions of cachexia in chronic illnesses are required to reflect specific nuances associated with each disease. These discrete cachexia definitions help with the precision of research and the subsequent clinical interventions to improve outcomes for patients suffering from cachexia. The absence of a definition for cachexia in an ESKD population makes it particularly difficult to study the incidence of cachexia or potential treatments, as there are no standardised inclusion criteria for patients with ESKD who have cachexia. Outcomes from this study will provide much needed data to inform development and testing of potential treatment modalities, aimed at enhancing current clinical practice, policy and education.

## Background

Cachexia is “*a complex metabolic syndrome associated with underlying illness and characterised by muscle loss, with or without loss of fat*” [8]. It is a common syndrome associated with chronic illness and has been defined in the general population as weight loss of at least 5% in ≤12 months or body mass index (BMI) < 20 kg/m^2^ plus three of the following five features: decreased muscle strength; fatigue; anorexia; low fat-free mass index; abnormal biochemistry (increased inflammatory markers [C-reactive protein (CRP) > 5 mg/L], anaemia [haemoglobin < 120 g/L] and low serum albumin [< 32 g/L] [8]. To date, research has not explored the impact of cachexia in end-stage kidney disease (ESKD) but studies into cachexia in other chronic illness have demonstrated the devastating holistic impact it can have on patients, decreasing physical function and quality of life [26] and shortening survival [9, 30].

Research within a haemodialysis ESKD population has established that weight stable patients survive longer than those with weight loss [28]. Additionally, it is known that wasting/cachexia occurs in up to 75% of ESKD patients on haemodialysis [14]. However, despite the high prevalence of cachexia in haemodialysis patients there has been relatively little research specifically targeted to this at risk population] [14]. For “renal cachexia” there are no standardised definitions or inclusion criteria to help inform practice or research [25]. Thus, the clinical management of cachexia in persons with ESKD is challenging [14]. In part, this is due to the difficulty discriminating cachexia from other causes of malnutrition and weight loss [23]. For persons with ESKD there is now greater emphasis on defining clinical markers for Protein Energy Wasting (PEW) which precedes cachexia and specialised diagnostic tools are being developed and tested [5, 16] Cachexia is seen as a severe form of PEW [11, 13], however it is important to be able to clinically differentiate between cachexia and PEW as each may require distinct management strategies; such discrimination may also be important when defining target groups for future trials of novel pharmacological or nutritional interventions [21].

In summary, there remains limited consensus on the defining characteristics of renal cachexia partly due to limited epidemiological research on this syndrome in ESKD. Although there is improved understanding of the mechanisms underpinning cachexia in ESKD [27], further research in this area and planning targeted clinical interventions is hampered by the absence of an accurate disease-specific cachexia phenotype in ESKD [7]. A generic definition of cachexia in chronic illness is available [8] but the associated diagnostic criteria are not specific to ESKD. The necessity to re-model this definition in specific diseases is well recognised within the literature ([10]: [14]). Indeed, where this has been done in cancer, it has led to a consensus definition and classification system [1, 10], clinical practice guidelines [20] and has made a positive impact to clinical management, research study design, policy and also patient and professional education. There is a pressing need to robustly define the clinical phenotype of renal cachexia to help target supportive measures for this population [6, 17].

## Methods/design

### Aim and objectives

The aim of this study is to determine the clinical phenotype of cachexia specific to individuals with ESKD.

The objectives of the study are to:

- Identify the prevalence of cachexia in ESKD patients receiving haemodialysis
- Determine the percentage of ESKD patients receiving haemodialysis who experience the known characteristics of cachexia (how many and to what degree)
- Develop a definition of cachexia specific to this ESKD population.

### Study population

This is a longitudinal study which will run over 2 years. All patients with ESKD who receive  haemodialysis and attend a single Regional Nephrology Unit within the United Kingdom, will be eligible for inclusion into this study to determine if they experience (and to what degree) the known characteristics associated with cachexia (Table 1). Key factors, such as: primary renal disease; comorbidities (eg Charlson Comorbidity Index); recent hospitalisation (within 3 months); drugs (particularly steroids, erythropoietin stimulating agents and immunosuppressive agents); urea clearance; residual renal function; and dialysis vintage will also be recorded.

**Table 1:** Characteristics of Cachexia [8, 14]

Unintentional oedema free weight loss ≥ 5% in 6 months or ≥ 10% 12 months (in the absence of documented weights, BMI < 20 kg/m^2^) and in the absence of starvation.
Plus three of the following five criteria:
1) Decreased muscle strength(lowest tertile);
2) Fatigue (physical and/or mental weariness resulting from exertion not merely post-dialysis fatigue);
3) Anorexia;
4) Lean tissue depletion (in relation to aged /gender normative data)
5) Abnormal biochemistry: increased inflammatory markers [CRP > 5 mg/L]; anaemia [haemoglobin < 120 g/L]; low serum albumin [< 32 g/L bromocresol green].

The recruiting regional nephrology unit provides care to approximately 250 chronic haemodialysis patients per annum. Recruitment will take place over 7 months, with an expected recruitment rate of 145 patients. Suitable patients will be identified via inpatient wards and outpatient units at the recruiting regional nephrology unit. Inclusion/exclusion criteria are outlined below. Once recruited patients will be monitored for 1 year (or time to death if < 1 year).

### Data collection

**Fig. 1:**
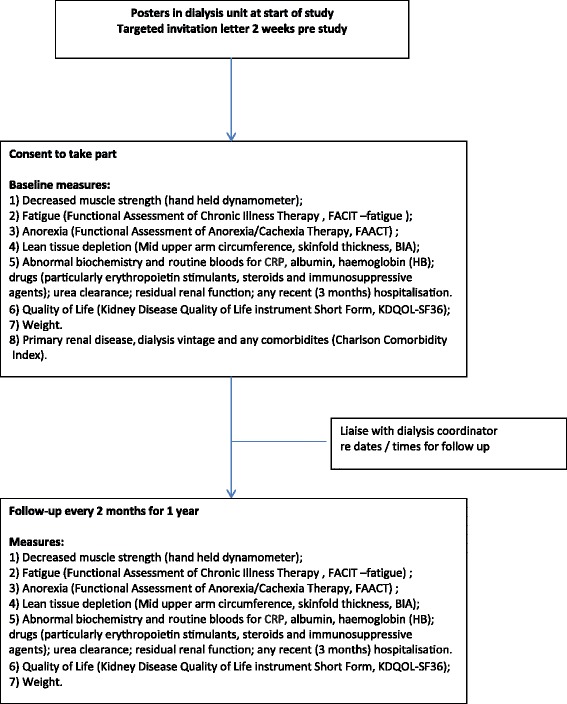
Recruitment and follow up diagram

This study is ‘ongoing’ and is currently carrying out recruitment. All measures will be assessed at baseline and will be repeated every 2 months for 12 months or until death (Fig. 1). Patients will be asked to complete validated tools to determine fatigue, quality of life and anorexia. We will measure: weight; lean muscle mass (bioelectrical impedance analysed via vector analysis, mid upper arm muscle circumference - from measures of mid arm circumference and tricep skin fold thickness); muscle strength (hand held dynamometer); and interrogate routine monthly clinical blood tests for abnormal biochemistry (Table 1).

Any dialysis unit is a busy clinical environment and patients attending for haemodialysis three times per week do not want to be delayed in entering or leaving the dialysis unit. Thus it is imperative that the study does not over burden the clinical area or participants. We have worked closely with the clinical team to plan how we will collect data from patients so as not to interrupt the clinical flow or delay the patient unnecessarily when they have completed a dialysis session. Where possible routinely collected clinical data will be used e.g. pre- and post-dialysis blood samples, weight pre- and post-dialysis. We will ask patients (or help patients) to complete the tools measuring quality of life, fatigue and anorexia while on dialysis and we will then measure hand grip strength, mid upper arm circumference, skin fold thickness and complete Bioelectrical Impedance Analysis (BIA) immediately post-dialysis. It is expected these assessments with take 15 min to complete. Inclusion criteria requires a confirmed diagnosis of stage 5 CKD (estimated GFR <15mL/min/1.73m^2^) receiving dialysis, over the age of 18 and able to read and write.  Exclusion criteria involves those who are Stage 1-4 CKD, Stage 5 CKD who are not receiving haemodialysis, lacking capacity to give consent, under the age of 18 or non-english speaking. 

### Study withdrawal

Participants will be advised of the voluntary nature of their inclusion in this research and can withdraw from follow-up at their request. As stated within the information sheet, for any patient who does withdraw, we will use collected data up to the withdrawal point (with the participant’s consent) and this may be used for analysis. We will record all reasons for patient withdrawal during the data collection of this study.

### Instruments

Within this study we will use the World Health Organization’s (WHO) definition of quality of life which is “*individuals’ perception of their position in life in the context of the culture and value systems in which they live and in relation to their goals, expectations, standards and concerns*”. The tool we will use to measure quality of life will be the Kidney Disease Quality of Life instrument Short Form- KDQOL-SF36 [12]. This tool has been selected as it has confirmed reliability and validity in measuring quality of life in an end stage renal disease population [2]. The FACIT-fatigue scale, will be used to measure fatigue. It has proven psychometrics and is time efficient to complete and suitable to use for the purposes proposed [4]. We will also measure anorexia using the Functional Assessment of Anorexia/Cachexia Therapy (FAACT) questionnaire which is a validated appetite assessment tool for patients receiving dialysis [15].

### Analysis

This is a small scale longitudinal study. The data collected will provide information on ESKD participants who do and do not exhibit known characteristics of cachexia. The goal is to recruit and follow a population of ESKD patients who are receiving haemodialysis for 1 year (or until death) to identify those who exhibit characteristics of cachexia, to what degree, and to compare them to those without cachexia. The findings from this study will inform a disease-specific definition for cachexia in the ESKD population. Summary statistics will be calculated regarding the characteristics of participants. Analysis will be done comparing combinations of criteria (Table 1) and their outcomes using multivariate logistic regression and compare time to death with an age and sex matched parallel cohort of ESKD participants receiving haemodialysis without weight loss and associated characteristics of cachexia. Analysis of raw bioelectrical impedance data will be undertaken to estimate nutritional status and presence of a cachexia phenotype. Conversion of impedance readings into fat free mass, raw analysis by bioelectrical impedance vector analysis and bioelectrical impedance phase angle assessment will be undertaken. The longitudinal data collected will be analysed to develop a model potentially based on mortality and which factors best associate with this. This will then be prospectively tested in future studies.

### Ethical considerations

This study will be conducted in compliance with Good Clinical Practice Guidelines. Governance approval for the study has been obtained from the host institution and the Office of Research Ethics Committees Northern Ireland (ORECNI) approval has been gained in preparation for the study commencing (REC reference: 16/NI/0233). Fundamental principles of good practice including the provision of user friendly information sheets, informed consent, voluntary participation, confidentiality and data protection procedures will be applied as a minimum standard within this study. Professional gatekeepers will establish primary contact with potential participates about this study. It is acknowledged that participants may find completion of questionnaires distressing and a distress protocol has been designed to manage such distress. The study is designed so that it will not be over burdensome for participants. For example, the validated tools can be completed whilst on dialysis, we will access routine clinical information were we can (bloods and weight post dialysis). All measures of lean muscle mass will be collected post-dialysis and we will adhere to the manualized techniques for the instruments used.

### Patient and public involvement (PPI) in this research

Members of the research team are also members of the Northern Ireland Kidney Patient Association (NIKPA) (JR, HN, PM, JS), Northern Ireland Kidney Research Fund (PM, JS) and the British Kidney Patient Association (HN). The NIKPA hold monthly meetings and are in regular e-mail contact with members. The programme of renal cachexia work has been discussed since inception with the NIKPA and had been presented to their members, by the lead researcher (JR), facilitated by an NIKPA member. The Northern Ireland Kidney Research Fund fully support the planned research and members have been able to discuss the proposal with the study team and have made practical suggestions to improve its design, such as the use of the term cachexia in patient facing material. Members of these organisations have actively contributed to the study proposal and have been influential in shaping its scope. Our patient and public involvement organisations were represented at our September 2014 “Defining Renal Cachexia” workshop led by the authors. A British Kidney Patient Association representative was a speaker at this workshop. Two NIKPA members were attendees and actively engaged with workshop discussions.

## Discussion

From this research we will identify the characteristics of cachexia in an ESKD population receiving haemodialysis. This predictive profile will require prospective validation, which will be done through further research with a larger population. The findings from this study will be used as pilot clinical data to inform a larger multi-site study to develop a consensus definition for ESKD patients who have cachexia and test a treatment modality aimed at improving morbidity and mortality. Establishing a clinical phenotype and in turn a consensus definition for renal cachexia will allow for early recognition and identification of the syndrome. Developing a consensus definition will allow future research to be designed which aims to develop and test targeted interventional strategies aimed at improving both quality of life and morbidity in patients on haemodialysis. This in turn will help to standardise holistic care to this client group through the development of patient centred care guidelines and patient care pathways. It is acknowledged that research with palliative populations faces particular challenges as the patient’s condition deteriorates. The research team has extensive experience working with such populations [18, 19, 23, 24].

In planning this study the research team has worked closely with clinical colleagues to ensure that: data collection points will coincide with planned dialysis; and routine clinical data already available (such as monthly blood tests performed in all haemodialysis patients as part of their standard care) will be used to minimize any potential additional burden to patients. We do expect deaths within the sample population; most recent data (2010–2015) from the recruiting regional nephrology unit, indicates an annual mortality of 16% for patients with ESKD receiving maintenance haemodialysis.

Cachexia within a renal population may be an indicator of poor prognosis, as is the case in other chronic illnesses [3]. A robust definition of cachexia in ESKD is a pre-requisite for researchers and clinical teams to allow them to study and test interventions that can improve both the quality of life and survival of individuals with this condition.

## Conclusions

There is no doubt that the clinical management of cachexia in persons with chronic kidney disease is challenging due to the polysymptomatic nature of cachexia. However to date, limited attention has been devoted to cachexia in end stage kidney disease [14, 29]. For renal cachexia, there are no standardized definitions or inclusion criteria to help inform practice or research [25]. The importance of identifying the disease specific key features of cachexia is evident in the cancer population, where such work has allowed the biopsychosocial impact of this syndrome to be researched and potential therapeutic inventions trialled [22].

## Abbreviations

ESKD: End-stage kidney disease
NIKPA: Northern Ireland Kidney Patient Association
ORECNI: Office of Research Ethics Committees Northern Ireland
PPI: Patient and Public Involvement
